# Perceptual compensation for differences in speaking style

**DOI:** 10.3389/fpsyg.2013.00399

**Published:** 2013-07-02

**Authors:** A. Davi Vitela, Natasha Warner, Andrew J. Lotto

**Affiliations:** ^1^Speech, Language and Hearing Sciences, University of ArizonaTucson, AZ, USA; ^2^Department of Linguistics, University of ArizonaTucson, AZ, USA

**Keywords:** speech perception, context effects, reduced speech, natural speech, auditory perception

## Abstract

It is well-established that listeners will shift their categorization of a target vowel as a function of acoustic characteristics of a preceding carrier phrase (CP). These results have been interpreted as an example of perceptual normalization for variability resulting from differences in talker anatomy. The present study examined whether listeners would normalize for acoustic variability resulting from differences in speaking style within a single talker. Two vowel series were synthesized that varied between central and peripheral vowels (the vowels in “beat”–“bit” and “bod”–“bud”). Each member of the series was appended to one of four CPs that were spoken in either a “clear” or “reduced” speech style. Participants categorized vowels in these eight contexts. A reliable shift in categorization as a function of speaking style was obtained for three of four phrase sets. This demonstrates that phrase context effects can be obtained with a single talker. However, the directions of the obtained shifts are not reliably predicted on the basis of the speaking style of the talker. Instead, it appears that the effect is determined by an interaction of the average spectrum of the phrase with the target vowel.

## INTRODUCTION

One of the central challenges for theories of speech perception has always been the infamous variability in the acoustic realization of phonemes. Much of the traditional focus has been on variability across talkers, due to differences in anatomy, physiology, and dialect, which listeners must accommodate to correctly categorize phonetic segments. There are a number of models for reducing this variability (particularly with vowel sounds) that rely on relationships between acoustic parameters (such as the formants and fundamental frequency) within the phonetic segment (Miller, 1989; Katz and Assmann, 2001; Smith et al., 2005); so-called *intrinsic* models (Ainsworth, 1975; Nearey, 1989). However, it is also clear that listeners must use information in the surrounding speech *extrinsic* to the target segment to accommodate the peculiarities of the talker (Joos, 1948; Ladefoged and Broadbent, 1957; Nearey, 1989). The importance of this extrinsic information has become even more apparent with recent demonstrations that listeners will shift their perception of vowel tokens depending on the dialect or accent of a talker (e.g., Evans and Iverson, 2004; Maye et al., 2008) or even idiosyncratic productions specific to an individual talker (Norris et al., 2003). In these experiments, listeners presumably learn something about typical phonological/phonetic patterns of the talker during a preceding phrase or set of phrases and alter their perception based on these expectations.

Whereas the variability associated with physiological and linguistic differences between talkers is salient, substantial complexity is present in the acoustic–phonemic mapping even within a single talker. Depending on the communicative setting, talkers will vary their speech style from clear, precise productions to less energetic, reduced productions. Reduced productions are those that are typical of conversational speech, typified by deletions of segments or productions that are not articulated in their canonical form (Johnson, 2004; Pluymaekers et al., 2005; Warner, 2005; Nakamura et al., 2007). As codified in Lindblom’s (1990) H&H theory, this shift from Hyper to Hypo-speech is a result of an interaction between the need to provide clear information and the desire to minimize effort. The relative balance between these constraints varies from conversation to conversation and even within a single discourse. According to Lindblom (1990, 1996) this variation of speech style is the result of an adaptive communication system. However, robust communication can only be maintained if the listener can accommodate the acoustic variability arising from these shifts in speaking style.

There has been quite a bit of work characterizing the acoustic changes that accompany shifts in speaking style. In particular, there has been an in-depth description of the results of a talker trying to produce “clear” intelligible speech (e.g., Picheny et al., 1986; Moon and Lindblom, 1994; Bradlow et al., 2003; Lotto et al., 2006). The consequences of clear speech production include increased segment durations, greater f0 ranges, and shifts in vowel formant frequencies resulting in an expanded vowel space (Chen, 1980; Picheny et al., 1986; Moon and Lindblom, 1994; Ferguson and Kewley-Port, 2002; Bradlow et al., 2003). It should be noted that in most of these studies the comparisons are between very “clear” speech, such as one may produce when speaking to an interlocutor who does not share the native language, and the relatively clear speech that is normally produced in laboratory recordings. That is, much of the research has been conducted on the “hyper” end of Lindblom’s H&H continuum. Recently, there has been a growth in interest in hypo-articulated, reduced speech (e.g., Greenberg, 1999; Ernestus, 2000; Johnson, 2004; Warner et al., 2009).

Whereas the textbook descriptions of how acoustic cues such as formant frequencies map onto phonetic contrasts are based largely on the speech style of elicited careful productions, it is probable that listeners’ perceptual categorization of speech sounds must adapt to the substantial variability in form across speech styles. For example, the same F1 and F2 frequencies of a vowel could be the result of a hypo-articulated /i/ (vowel sound in “beat”) or a hyper-articulated /I/ (vowel sound in “bit”). In fact, when vowels or words are cut from a stream of conversational speech and presented in isolation, listeners perform rather poorly in recognition tasks (e.g., Koopmans-van Beinum, 1980). But, when the initial context is preserved, accuracy improves dramatically (Arai, 1999; Ernestus et al., 2002). Further, listeners perform better when the context is presented acoustically, rather than visually, demonstrating that the syntactic/semantic information alone is not as beneficial as when it is provided with the acoustic information (Janse and Ernestus, 2011). Recent evidence has proposed that listeners’ knowledge of typical patterns of reduction and speaking rate affect their perception of subsequent phonemes or words (Mitterer and Ernestus, 2006; Mitterer and McQueen, 2009; Dilley and Pitt, 2010). That is, listeners appear to compensate for speech reduction.

One possible explanation for the facilitative nature of context is that the listeners are “tuning” their phonemic categories based on expectations derived from the context. Perhaps listeners can gauge the talker’s speaking style along the H&H continuum and use this to drive expectations for phonetic realizations. A target vowel that is surrounded by a hyper-articulated context is likely to be close to its canonical formant values (for that talker, Lindblom, 1996). Recognizing the speaking style and making comparisons based on that recognition would be similar to recognizing an accent or dialect and shifting the perceptual space (or the exemplars/prototypes to which one is making comparison) based on previous experience with that accent (e.g., Evans and Iverson, 2004). Alternatively, listeners could keep a running map of the current vowel space produced by the talker in the context without explicitly noting the speaking style and use this map to determine the likely identity of the target vowel. An ambiguous /i/–/I/ vowel may be categorized as /I/ in comparison to the expanded vowel space typical of clear speech (where the formants of the vowel will be closer to the center of the space as is typical for an /I/) but as /i/ when surrounded by a context with a reduced vowel space (where the same formants will be closer to the periphery of the space as typical for an /i/).

The proposal that contextual information can be used by the listener to tune their phonetic categorizations is reminiscent of the classic work by Ladefoged and Broadbent (1957) on perceptual normalization of acoustic differences between talkers. The problem of inter-talker variability is similar to that described above for intra-talker variability – a particular F1 frequency could be appropriate for /ε/ (vowel sound in “bet”) produced by one talker or could be /I/ (vowel sound in “bit”) produced by a talker with a smaller vocal tract (or /I/–/i/, respectively, from our previous example). In order to examine how listeners dealt with this ambiguity, Ladefoged and Broadbent (1957) presented a target word /bVt/ following a context carrier phrase (CP; “Please say what this word is…”). The target was kept constant but the CP was manipulated by decreasing the F1 frequencies of the vowels (this was one of a number of manipulations that were examined), which may be conceived as a talker with different anatomy or idiosyncratic vocal tract positioning. Following the lower-F1 context, listeners changed their categorization of the target from *bit* to *bet*. This result could be due to listeners extracting general information about the talker’s production style (e.g., that they hold their tongue in a relatively high position throughout) or by mapping a vowel space for the talker from the lexical–acoustic information in the context and finding the relative position for the target vowel. Whether listeners are extracting general or vowel-specific information from the context, it would appear that these same talker normalization mechanisms could also be available to allow the perceptual accommodation of speaking style variability within a talker.

The main goal of the set of experiments described below was to determine if listeners do, in fact, shift their categorizations of target speech sounds as a result of changes in speaking style of a context phrase using a methodology similar to that used by Ladefoged and Broadbent (1957). Unlike Ladefoged and Broadbent (1957), though, our CPs were all produced by the *same* talker to explicitly test whether changes in speaking style elicited an effect on target perception. Thus, these experiments are also a test of whether classic “talker normalization” effects are applicable to variability arising from the speech of a single talker. In particular, we used two target vowel contrasts – /i/ versus /I/ (vowel in “beat” versus vowel in “bit”; Experiment 1) and /α/ versus /Λ/ (vowel in “bod” versus vowel in “bud”; Experiment 2). Each pair contains a member that sits on the periphery of the vowel space and a member that is located more central. If listeners do “tune” their vowel categorization to the speaking style of the talker, then we predict that ambiguous vowel tokens will be perceived as more central (/I/ and /Λ/) in the context of clearly produced phrases (with an expanded vowel space) and as more peripheral (/i/ and /α/) following casual-reduced productions of the same phrases (with a more centralized vowel space). That is, the listener will categorize the targets relative to expectations of acoustic realizations derived from the speaking style of the preceding phrases.

## EXPERIMENT I

In order to test whether differences in speaking style can affect listener vowel categorizations, participants were asked to identify members of a synthesized vowel series that varied from /i/ (more peripheral) to /I/ (more central) that were appended to the end of a CP. Each CP was produced with a “clear” and “reduced” speech style by the same talker. Effects of speaking style are predicted to result in significantly more /i/ responses when the targets follow a CP produced with a reduced (hypo) speaking style.

### MATERIALS AND METHODS

#### Participants

Thirteen undergraduate students were recruited from the University of Arizona, received course credit for their participation, and provided informed written consent before participating in the experiment. All reported normal hearing and English as their native language.

This experiment was approved by the Institutional Review Board at the University of Arizona.

#### Stimuli

**Carrier phrases.** Two CPs were recorded by a female trained phonetician with experience producing both clear and reduced speech (Natasha Warner). The sentences were: “Please press the key that matches what I say” (CP1) and “Please would you choose the label most like the vowel I say” (CP2). The first sentence was created to contain mostly front vowels and no non-low back vowels in order to provide a better sampling of the vowel space around the target series. In this way, it is similar to “Please say what this word is” of Ladefoged and Broadbent (1957) which includes more front vowels. The second sentence was created to provide a better sampling of the entire vowel space, especially the most peripheral vowels. Each sentence was recorded four times in both reduced and clear speech styles. Recordings were made digitally with a 44.1-kHz sample rate, which was later converted to 22.05-kHz to match the appended target stimuli. The authors then chose one exemplar of each phrase/style that best exemplified the clear/reduced distinction. PRAAT (v.5.1.25; Boersma and Weenik, 2010) was used to calculate the average values of f0 and the first three formants across each CP. These average values, and the durations of each CP, are shown in **Table 1**. The reduced phrases were shorter than the clear productions as expected from previous acoustic analyses of speech style (e.g., Picheny et al., 1986; Bradlow et al., 2003).

**Table 1:** Duration of carrier phrases in seconds and average fundamental frequency, F1, F2, and F3 in both reduced and clear speaking styles.

Carrier phrase	Time (s)		f0 (Hz)		F1 (Hz)		F2 (Hz)		F3 (Hz)	
	CLEAR	REDUCED	CLEAR	REDUCED	CLEAR	REDUCED	CLEAR	REDUCED	CLEAR	REDUCED
CP 1	2.50	1.52	165	173	878	985	2351	2304	3407	3420
CP 2	4.10	2.35	171	169	815	857	2168	2047	3478	3426
CP 3	2.18	1.31	175	161	961	920	2222	2107	3596	3525
CP 4	0.97	.67	187	168	826	799	1941	1780	3154	3257

**Targets.** An /i/–/I/ target series was synthesized (with a 22.05-kHz sampling rate) using a version of the Klatt synthesizer (Klatt, 1980). The synthesizer parameters used for the endpoint stimuli were determined from acoustic measures of clear /i/–/I/ stimuli produced by the same talker who provided the CPs. Four productions of /i/ and /I/ in a /hVd/ context were digitally recorded, the best exemplars were chosen, and their formant frequency values were measured at vowel midpoint. The endpoint frequency values for formants 1 through 4 are presented in **Table 2** along with the step sizes in values between each member of the series in order to create 10 target vowels. Duration was fixed at 100 ms and f0 was 180 Hz (the average f0 for /hVd/ productions by this talker).

**Table 2:** Formant values and step changes to create the vowel series (/i/ to /I/ and /Λ/ to /α/) with the Klatt synthesizer.

Series 1	/i/	Step value	/I/
F1	360	+12	480
F2	2670	-31	2360
F3	3460	-35	3110
F4	3500	–	3500
**Series 2**	**/Λ/**	**Step value**	**/α/**
F1	730	+13	860
F2	1520	-10	1420
F3	2840	+3	2870
F4	3500	–	3500

**Carrier phrase + target.** The CPs were all matched in average intensity (equated root means square, RMS) and the targets were +3 dB higher than the average intensity. Each target was digitally appended to each of the four CPs following 50 ms of silence using Adobe Audition (Balo et al., 1992–2004) for a total of 40 stimuli (2 CPs × 2 speech styles × 10 targets).

#### Procedure

Participants were run in groups of one to three at a time on separate computers in a quiet room. Before starting the study, the experimenter explained to each group that their task was to identify the vowel sound that they heard at the end of the phrase. The experimenter played the vowel series and several of the CPs + targets (endpoints only) so that participants would be familiar with how the stimuli sounded and could ask any questions that they had.

On each trial, participants were presented a single randomly-selected CP + target stimulus over circumaural headphones (Sennheiser HD 280) at approximately 75 dB sound pressure level (SPL). They categorized the target vowel by using a mouse to click one of two boxes on a monitor labeled “beat” and “bit” (they were told that the targets matched one of the vowels in these words). The next trial would not begin until the participant responded. Two blocks of five repetitions of each stimulus (for a total of 400 trials) were presented and participants were offered a short break between blocks. The entire session took approximately 30 min. Stimulus presentation and data collection were controlled by the ALVIN software program (Hillenbrand and Gayvert, 2005).

### RESULTS

**FIGURE 1:**
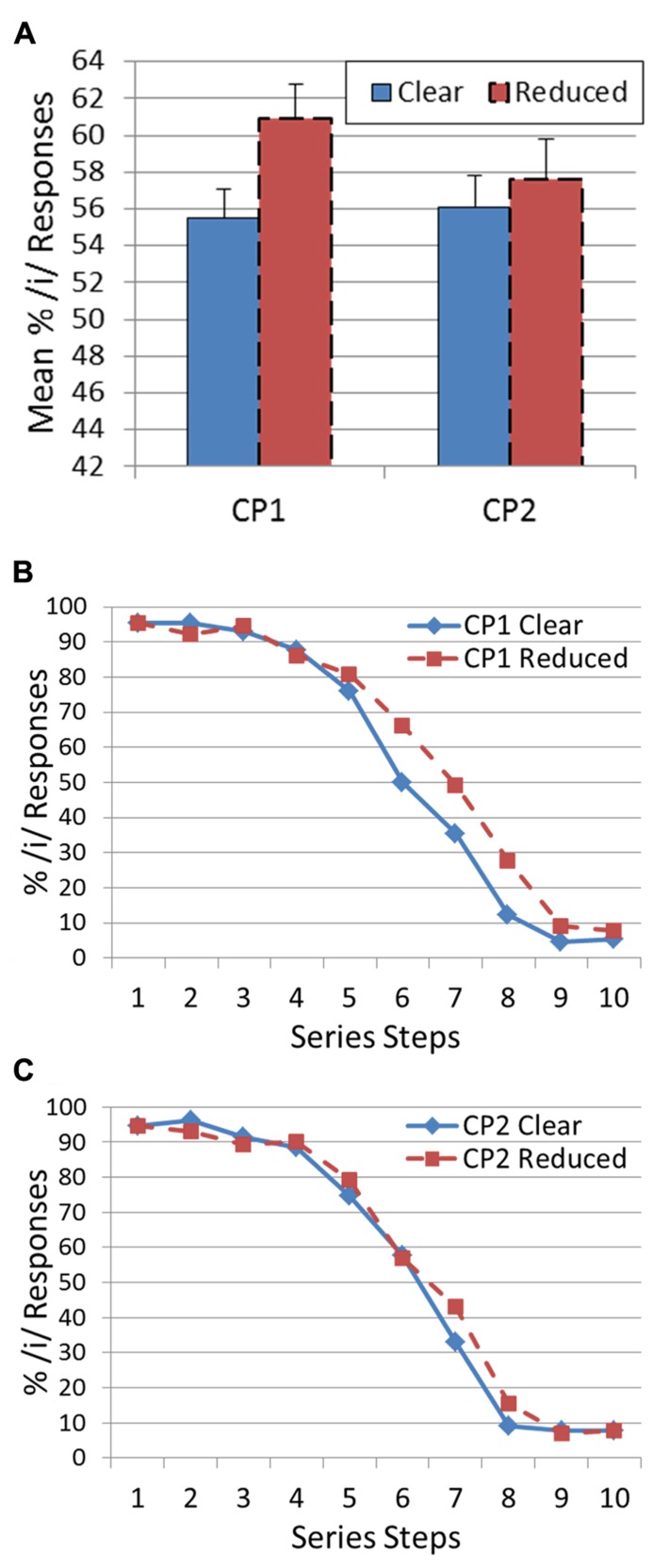
** Results of Experiment 1: (A) Histogram showing mean percentage of /i/ responses for CP1 and CP2 for both a clear and reduced speaking style.** Error bars indicate standard error of the mean. **(B)** Categorization function showing mean percentage of /i/ responses across vowel series from /i/ to /I/ for CP1. Speaking style is represented by a different line. **(C)** Categorization function showing mean percentage of /i/ responses across vowel series from /i/ to /I/ for CP2. Speaking style is represented by a different line.

The prediction is that if listeners tune their vowel categorization to the talker’s speech style, they will respond /i/ more often when the target is preceded by a reduced-style CP. This is because ambiguous targets between /i/ and /I/ are more likely to be reduced versions of /i/ (as opposed to hyper-articulated versions of /I/). To test this prediction, separate paired-sample *t*-tests were conducted for CP1 and CP2 with percent of /i/ responses collapsed across all series members serving as the dependent variable and speech style (clear versus reduced) serving as the independent variable. For CP1, there was a significant shift in /i/ responses [*t*(12) = 2.72, *p* < 0.05] in the predicted direction (clear: 55.5%; reduced: 60.9%). On the other hand, there was no significant shift in vowel categorization [*t*(12) = 0.90, *p* = 0.39] for CP2 (clear: 56.1%; reduced: 57.6%). **Figure 1** presents the histograms and categorization functions for the targets as a function of the CP speaking style for CP1 and CP2.

The results for CP1 provide evidence that vowel categorizations can be significantly shifted by variations in speaking style. This result extends the demonstrations of perceptual shifts from CPs produced by presumably different talkers by Ladefoged and Broadbent (1957) and subsequent researchers (e.g., Johnson, 1990; Watkins and Makin, 1994) to phrases that are clearly produced by the same talker using a different style. However, the lack of a similar shift for CP2 raises the question of whether this result is particularly generalizable (a question that has also been raised about previous between talker effects – Dechovitz, 1977). In order to test whether this effect is robust, we attempted to replicate the study using a second vowel contrast and different CPs.

## EXPERIMENT II

### MATERIALS AND METHODS

#### Participants

Ten undergraduate students were recruited from the University of Arizona, received course credit for their participation, and provided informed written consent before participating in the experiment. All reported normal hearing and English as their native language. None had participated in Experiment 1.

This experiment was approved by the Institutional Review Board at the University of Arizona.

#### Stimuli

**Carrier phrases.** Two new CPs were constructed in order to emphasize the central/low region of the vowel space near the target vowel contrast – /α/ versus /Λ/: “Touch the button for what comes up” (CP3) and “Abracadabra” (CP4). “Abracadabra” was chosen to match other work with an articulatory synthesizer that is constrained in the phrases that it can produce (Vitela et al., 2009) and because it fit the requirements of Experiment 2 (central/low vowels). These were recorded by the same speaker using the same set-up as for the phrases in Experiment 1.

**Targets.** The 10-step target vowel series was synthesized to vary from /α/ (peripheral) to /Λ/ (central). The endpoints were again based on /hVd/ productions by our speaker and the formant values are presented in **Table 2**. All other details matched the target series utilized in Experiment 1. The appending of CP and target also followed the same procedure as in Experiment 1.

#### Procedure

The procedure was identical to Experiment 1 except that the response boxes were labeled “bod” and “bud.”

### RESULTS

**FIGURE 2:**
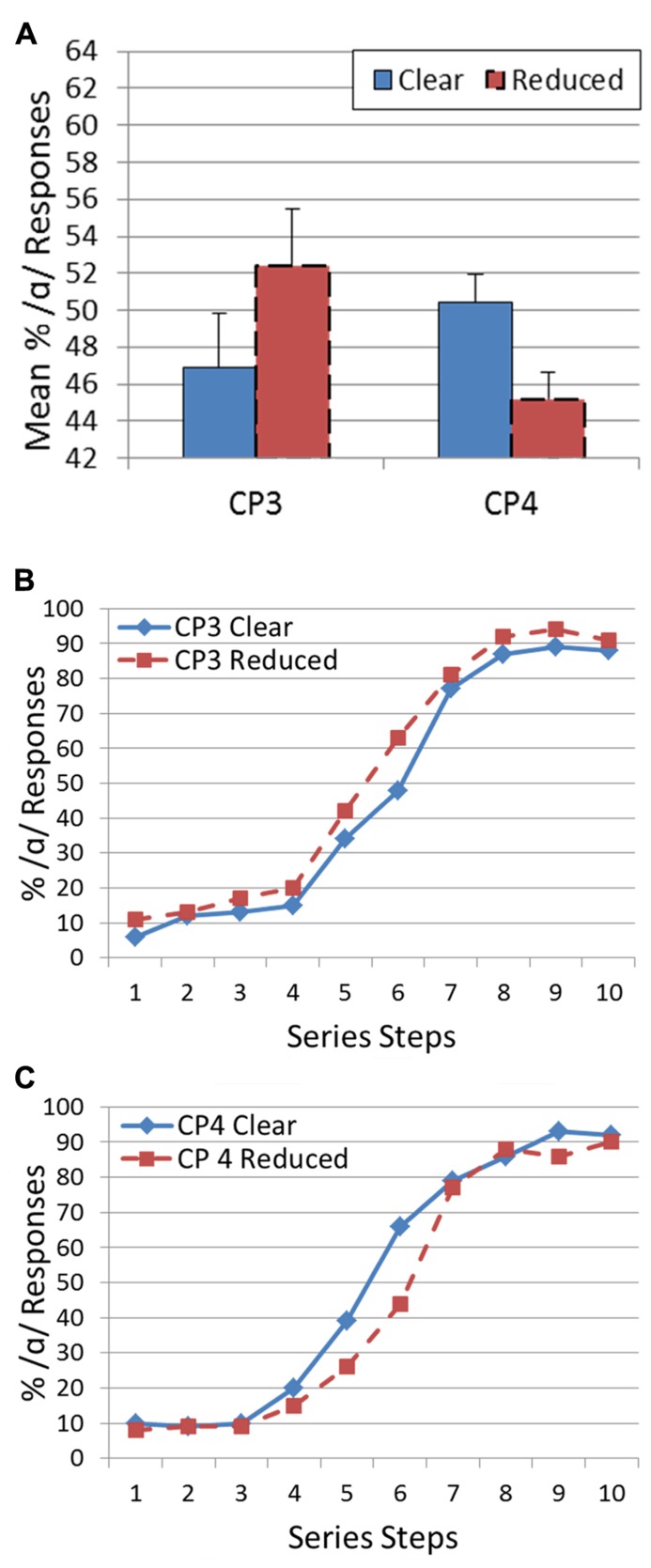
** Results of Experiment 2: (A) Histogram showing mean percentage of /α/ responses for CP1 and CP2 for both a clear and reduced speaking style.** Error bars indicate standard error of the mean. **(B)** Categorization functions showing mean percentage of /α/ responses across vowel series from /Λ/ to /α/ for CP3. Speaking style is represented by a different line. **(C)** Categorization functions showing mean percentage of /α/ responses across vowel series from /Λ/ to /α/ for CP4. Speaking style is represented by a different line.

Again, the prediction is that participants will respond /α/ more often (the more peripheral vowel) when the target follows a reduced version of each CP. For CP3, there was a significant shift in percent /α/ responses [*t*(9) = 2.58, *p* < 0.05] in the predicted direction (clear: 46.9%; reduced: 52.4%). Variation in speech style for CP4 (“Abracadabra”) also resulted in a significant target categorization shift [*t*(9) = 4.30, *p* < 0.005]. However, in this case the shift was in the opposite direction (clear: 50.4%; reduced: 45.2%). **Figure 2** presents the categorization functions for the targets as a function of the CP speaking style for CP3 and CP4.

Across Experiments 1 and 2, changes in speaking style of a CP resulted in significant shifts in target vowel categorizations for three out of four phrases. However, the direction of shift for CP4 complicates the explanation for these perceptual context effects.

## DISCUSSION

**FIGURE 3:**
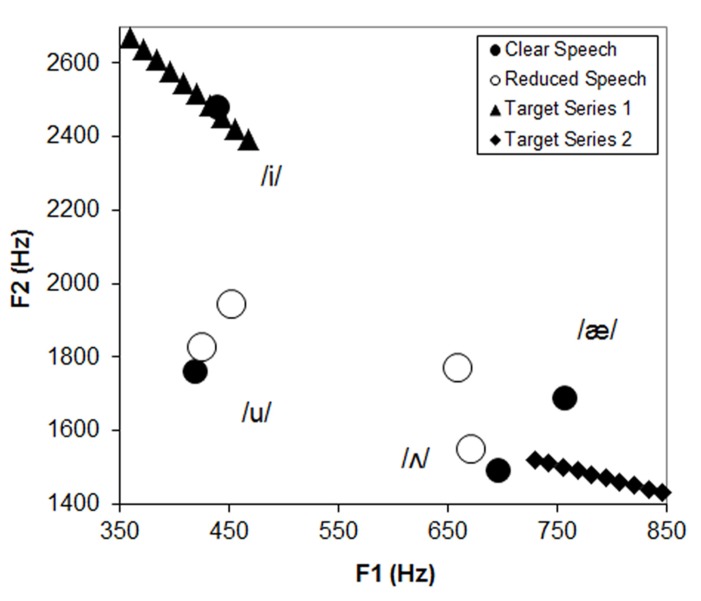
** Vowel space plotting the F1 and F2 values for four vowels selected from the carrier phrases in their clear and reduced forms along with values for target series**.

The experiments described above were designed to test whether listeners perceptually compensate for acoustic variations due to a talker’s speaking style using a paradigm that has been successful for demonstrating compensation for between-talker differences (Ladefoged and Broadbent, 1957). Members of two vowel contrast series (/i/–/I/ and /α/–/Λ/) were presented at the end of CPs that were either spoken with a clear or reduced/casual speaking style by the same talker. The prediction was that more /i/ and /α/ responses would be obtained following a reduced CP. The reasoning behind this was that reduced speech is hypo-articulated and, thus, ambiguous vowels would more likely be centralized versions of the peripheral vowels. This can be seen in **Figure 3**, which displays the F1–F2 values for four vowels selected from across the CPs in their clear and reduced forms along with the values for the target series. It can be seen that the vowel space is expanded in clear speech as has been previously described in acoustic analyses of speech styles (Chen, 1980; Picheny et al., 1986; Moon and Lindblom, 1994; Ferguson and Kewley-Port, 2002). Ambiguous members of the /i/–/I/ target series appear more peripheral or /i/-like when compared to the /i/ from the reduced phrase. As a result, a listener may adapt to these acoustic differences by categorizing more of the targets as /i/ when they have evidence that the talker is using a reduced speech style. Likewise, an ambiguous member of an /α/–/Λ/ series should seem more /α/-like when compared to the same centralized space. This kind of perceptual compensation could be due to the listener noticing that the talker is speaking casually or it could result from comparing the target explicitly with auditory representations of vowel exemplars used in the CP (such as /i/ or /Λ/).

Two of the four CPs show evidence for this type of style compensation. CP1 resulted in more /i/ responses and CP3 resulted in more /α/ responses when produced with a reduced style. In and of itself, these positive results are interesting because they demonstrate an effect of context on target categorization even when the CPs are clearly produced by the same talker. However, the other two CPs did not lead to qualitatively similar results. There were no significant effects of speaking style for CP2. Even more troubling, CP4 resulted in significantly *fewer* /α/ responses when produced with a reduced style, counter to predictions.

The differential effects of the CPs are not likely to be due to listeners not being able to perceive the speaking style of the listener in CP2 or misperceiving it for CP4. The distinction between clear and reduced productions was very clear for all of the phrases. As seen in **Table 1**, the predicted shortening in phrase duration for reduced productions was approximately the same ratio for all phrases and was in fact greatest for CP2, which resulted in no shift. Another possibility is that the style-based changes in the vowel spaces were different for the different CPs. This explanation also does not appear valid. The changes in vowel formants based on speaking style all were in the predicted direction.

If the disparity of results is not the result of perceived differences at the level of the talker or at the phonetic level, then perhaps the explanation resides at a more basic level. Perhaps differences in the overall spectral patterns of the CPs are responsible for the context effects and not anything particular to speaking styles or vowel spaces. Work by Holt (2005, 2006) provides a possible analogy to the present results. Holt (2005) presented listeners with a target series varying from /dα/ to /gα/ preceded by a “melody” of tones (70-ms each; 2.1 s for the entire melody) that varied in frequency. The actual “melody” varied randomly from trial to trial, as the tones were selected from a distribution. The main manipulation was the mean frequency of the distribution from which the tones were sampled. Participants labeled the target as /gα/ more often when the tones came from a distribution with a high mean frequency versus melodies with a low mean frequency. Laing et al. (2012) extended these findings to speech stimuli. Similar to Holt (2005), they presented listeners with tone sequences that preceded the /dα–gα/ targets. In addition, they used speech precursors that were manipulated to have peaks in frequency in different frequency ranges. They found that only those tone and speech precursors that had an average frequency or peak frequency within the third formant range had an effect on /dα–gα/perception. Those stimuli whose average or peak frequency was out of that range did not cause a shift in phoneme categorization. These effects were predicted because previous research had demonstrated that presenting increased energy (with either a speech or non-speech sound) in the frequency region slightly above the onset frequency of the third formant resulted in an effectively lower formant in the target – with /gα/ being the syllable with a lower F3-onset frequency (Lotto and Kluender, 1998; Lotto et al., 2003). What the results of Holt (2005, 2006) and Laing et al. (2012) suggest is that listeners compute a representation of the average spectral energy of preceding context and represent the target relative to that preceding average (so that high-frequency tones in a context result in a lower perceived F3 frequency in the target). This conclusion is supported by work by Watkins (1988, 1991) who applied a filter of a particular shape to a CP and demonstrated that a target vowel was perceived as if it were filtered with the inverse of the phrase filter (see also Watkins and Makin, 1994, 1996; Kiefte and Kluender, 2001). That is, listeners appeared to extract the filter shape from the phrase – which would be easiest from the averaged spectral envelope of the context – and then perceived the target vowel relative to it. Could differences in the average spectral representation of the CPs in the current experiment explain the shifts in target vowel identification?

**FIGURE 4:**
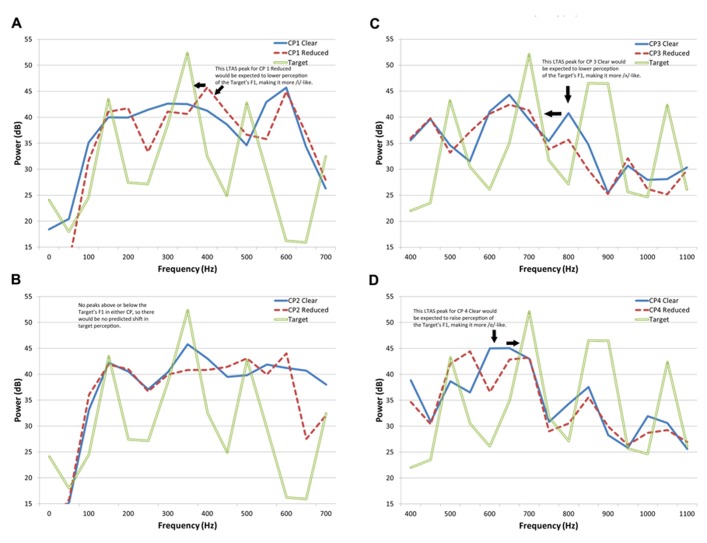
** Long-term average spectrum for each of the carrier phrases in each speaking style in the frequency range that includes F1 for the target stimuli.** **(A)** Carrier phrase 1; **(B)** carrier phrase 2; **(C)** carrier phrase 3; and **(D)** carrier phrase 4.

**Figure 4** presents the long-term average spectrum (LTAS) for each of the CPs in each speaking style for the frequency range that includes F1 for the target stimuli. The LTAS is the logarithmic power spectral density and can be thought of as the energy in frequency bands (here 50-Hz wide) across the entire phrase (the LTAS presented here were computed with PRAAT v.5.1.25; Boersma and Weenik, 2010). Plotted along with the CPs is the spectrum for the ambiguous target member that was at the boundary of the obtained categorization functions (step 6 from each target series). Because the effects of the context are greatest near the boundary region, this stimulus was used to make predictions of context LTAS on perceptual shifts. In **Figure 4A**, one can see the dominant peak of energy in the target that is a result of the F1 resonance. A peak in energy can also be seen just above (in frequency) this dominant peak for the reduced version of CP1. That peak is missing in the clear version of CP1. If one uses the Holt (2005) as an analogy, one would expect that the effective F1 would be perceived as lower frequency following the reduced phrase, resulting in a perception of a more /i/-like vowel (see Lotto and Holt, 2006). This is in fact the shift that was obtained. In contrast, one can see in **Figure 4B** that neither production of CP2 has a substantial peak directly above or below the peak in the target stimulus. In this case, no significant shift in responses was obtained. Thus, the relationships of the LTAS of the CPs and targets show some correspondence to the obtained results for Experiment 1.

In **Figure 4C**, one can again see the dominant energy peak related to target F1, this time for an ambiguous /α/–/Λ/ exemplar. There is also a peak in energy in the LTAS of CP3 present at a frequency above target F1 that is in one style but not the other. This time, however, it is present in the clear production. The result should be that the F1 is effectively lowered, which would this time lead to more /Λ/ responses (the response with a lower typical F1 frequency), which is what was obtained. Note that this is also the effect predicted from just considering the speech style of the talker. The obtained shift for CP4 was opposite this prediction. As seen in **Figure 4D**, the nearest phrase LTAS peak to the peak in the target spectrum is a peak for the clear phrase at a *lower* frequency. The prediction based on the results of Holt (2005), is that more /α/ responses (with a higher typical F1 frequency) will occur for the clear production. Again, this matches the results obtained.

It appears that the results obtained from all four CPs correspond qualitatively to what may be predicted from comparisons of target and context LTAS. It is possible that the same mechanism underlying the findings of Holt (2005) are responsible for the categorization shifts obtained here. Given that the contexts used by Holt (2005) were non-speech and similar types of context effects have been demonstrated in birds (Lotto et al., 1997), this responsible mechanism may actually be general auditory in nature, as opposed to being specific to speech or linguistic structure. This raises the question of the purpose of a general auditory process that computes LTAS to which new targets are compared. One suggestion that has been offered is that this process would provide noise reduction – by subtracting out continuous noise sources (which would show up in the LTAS) from transient acoustic changes that would contain information (Lotto and Sullivan, 2007).

It should be noted that although the results are qualitatively in line with an account of general auditory interactions that has been developed over the last decade, there is still no quantitative model of LTAS-based context effects. As such, it is difficult to assign a level of confidence that the account *predicts* the obtained findings. Given the difficulty of accounting for the effects from classical approaches to normalization (such as phonetic–acoustic mapping), the development of LTAS-based models may provide a viable and interesting approach to predicting perceptual-based accommodation of speech variability.

## CONCLUSION

Using the classic paradigm of Ladefoged and Broadbent (1957), we investigated whether listeners compensate for changes in speaking style. Lindblom’s (1990) H&H theory suggests that there is a continuum from very clear or hyper-articulated speech to very reduced or hypo-articulated speech. Speakers are thus flexible and adaptive in their production, balancing how clearly they need to speak to accurately convey a message with the minimal amount of effort necessary to do so. Thus, listeners must also be flexible and adaptive in their ability to perceive the message – whether it’s spoken clearly with high effort or casually with low effort. Listeners do this easily; yet, there is little understanding of the underlying mechanism. There is some suggestion that prior knowledge and experience with reduced forms and speaking rate allow listeners to compensate for a reduced speaking style (Mitterer and Ernestus, 2006; Mitterer and McQueen, 2009; Dilley and Pitt, 2010). It was proposed here that the prior context (CPs) could clue listeners in as to where they were listening on the H&H continuum, thus changing how they perceive subsequent productions (the target vowels). The results, however, did not all follow this prediction, but were more consistent with current models of local interactions that are of a general auditory nature (Vitela, 2012; Laing et al., 2012). However, these types of models have yet to be fully developed and so it is difficult to make strong claims of their explanatory power. There has been recent interest in extending the work on perceptual compensation for talker differences from those acoustic changes that can be the result of anatomical differences to those that could be due to dialects and accented speech (e.g., Dahan et al., 2008; Maye et al., 2008). As this work goes forward, it will be important to separate out those effects that are due to general auditory processing (such as LTAS-contrast effects) and those that are due to the changes in representations at the phonetic level.

## Conflict of Interest Statement

The authors declare that the research was conducted in the absence of any commercial or financial relationships that could be construed as a potential conflict of interest.
